# Lineage-Specific Real-Time RT-PCR for Yellow Fever Virus Outbreak Surveillance, Brazil

**DOI:** 10.3201/eid2311.171131

**Published:** 2017-11

**Authors:** Carlo Fischer, Maria C. Torres, Pranav Patel, Andres Moreira-Soto, Ernest A. Gould, Rémi N. Charrel, Xavier de Lamballerie, Rita Maria Ribeiro Nogueira, Patricia C. Sequeira, Cintia D.S. Rodrigues, Beate M. Kümmerer, Christian Drosten, Olfert Landt, Ana Maria Bispo de Filippis, Jan Felix Drexler

**Affiliations:** University of Bonn Medical Centre, Bonn, Germany (C. Fischer, A. Moreira-Soto, B.M. Kümmerer);; German Centre for Infection Research (DZIF) (C. Fischer, C. Drosten, J.F. Drexler);; Instituto Oswaldo Cruz, Rio de Janeiro, Brazil (M.C. Torres, R.M.R. Nogueira, P.C. Sequeira, C.D.S. Rodrigues, A.M.B. de Filippis);; TIB MOLBIOL Syntheselabor GmbH, Berlin, Germany (P. Patel, O. Landt);; Charité–Universitätsmedizin Berlin, corporate member of Freie Universität Berlin, Humboldt-Universität zu Berlin, and Berlin Institute of Health, Institute of Virology, Germany (A. Moreira-Soto, C. Drosten, J.F. Drexler);; Aix-Marseille University, Marseille, France (E.A. Gould, R.N. Charrel, X. de Lamballerie);; Institut Hospitalo Universitaire Méditerranée-Infection, Marseille (R.N. Charrel, X. de Lamballerie)

**Keywords:** yellow fever virus, surveillance, real-time RT-PCR, vaccine safety, vector-borne infections, Americas, Brazil, reverse transcription PCR, viruses

## Abstract

The current yellow fever outbreak in Brazil prompted widespread yellow fever virus (YFV) vaccination campaigns, imposing a responsibility to distinguish between vaccine- and wild-type YFV-associated disease. We developed novel multiplex real-time reverse transcription PCRs that differentiate between vaccine and American wild-type YFV. We validated these highly specific and sensitive assays in an outbreak setting.

Yellow fever virus (YFV) is a mosquitoborne member of the genus *Flavivirus* within the family *Flaviviridae* (Technical Appendix Figure 1, panel A) that is endemic to Africa and South America (*1*). Within YFV, 2 American and at least 3 African genotypes can be differentiated (*2*). The American YFV genotypes evolved from ancestral African viruses several hundred years ago and now are found only in South America (*3*).

In December 2016, Brazil reported the country’s largest yellow fever (YF) outbreak in decades. Through May 31, 2017, a total of 3,240 suspected cases were reported, including 435 deaths (*4*). The geographically widespread outbreak was caused by the American genotype 1 (Technical Appendix Figure 1, panel B) (*5*). In response to the outbreak, authorities launched large-scale vaccination campaigns aimed at distributing >20 million doses of YFV vaccine (*6*). Two different live-attenuated vaccines are being deployed. Most contain the vaccine strain 17DD, produced in Brazil (*7*). International authorities are deploying another 3.5 million doses of the standard vaccine strain, 17D (*6*). Both vaccine strains originate from the same parental strain, Asibi, and represent the West African genotype 2 (Technical Appendix Figure 1, panel B).

While YFV vaccines are considered safe, rare vaccine-associated adverse events (YF-VAAE) can occur (*8*). Viscerotropic YF-VAAE symptoms can overlap those of YF disease (*9*). In YFV-endemic regions, it is essential to distinguish between YF-VAAE and wild-type YFV infection (*10*). Routine diagnostic procedures can take several days, usually requiring 2 separate steps, detection and strain characterization by nucleotide sequencing. Here, we present 2 highly sensitive real-time reverse transcription PCRs (RT-PCRs) designed to detect and discriminate between YFV vaccine and American wild-type (hereafter referred to as wild-type) strains within 1 hour.

## The Study

**Figure 1:**
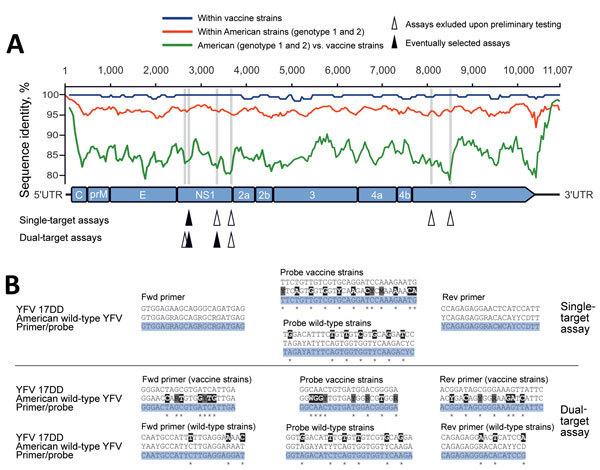
Design of new real-time RT-PCRs for differentiation between vaccine and wild-type YFV. A) YFV genomic representation (GenBank accession no. DQ100292) with real-time RT-PCR target sites, indicated by arrowheads, and identity plot of all complete YFV sequences available in GenBank as of May 24, 2017. Plots were done in SSE version 1.2 (*11*) using a sliding window of 200 and a step size of 40 nt. Target sites of the eventually selected assays are indicated by filled arrowheads; all other designed assays excluded after preliminary testing by open arrowheads. Of the real-time RT-PCR assays developed in this study, 1 assay targets only 1 genomic region, whereas the other assay targets 2 different genomic regions of vaccine and wild-type YFV strains. Both PCRs are duplex assays in which vaccine and wild-type YFV RNA are detected by lineage-specific probes. We called the assay targeting only 1 genomic region a single-target assay and the assay targeting 2 separate genomic regions a dual-target assay, even though the term dual-target commonly refers to detection of 2 different genes of a single pathogen, which is not the case in this study. B) Alignment of real-time RT-PCR oligonucleotide binding sites with YFV 17DD and American wild-type strains. The 100% consensus sequences were generated in Geneious (Biomatters Ltd., Auckland, New Zealand) and mapped to respective PCR primers and probes. Potential nucleotide mismatches are indicated by asterisks. D = A/G/T, M = A/C, R = A/G, W = A/T, Y = C/T. Black indicates a mismatch with all American wild-type strains, gray a mismatch with some American wild-type strains, based on the complete genetic information of American YFV strains and YFV vaccine strains available in GenBank as of March 24, 2017. C, capsid; E, envelope; Fwd, Forward; NS, nonstructural protein; prM, precursor membrane; Rev, reverse; RT-PCR, reverse transcription PCR; UTR, untranslated region; YFV, yellow fever virus.

We followed 2 rationales for real-time RT-PCR design. First, a small number of oligonucleotides per assay can be beneficial in resource-limited settings. Therefore, we designed 5 different single-target assays using primers capable of simultaneously amplifying vaccine and wild-type YFV strains. However, these criteria restricted the ability to design optimal oligonucleotides. Therefore, we designed 2 additional dual-target assays that target 2 separate genomic regions in which vaccine and wild-type strains differ sufficiently from one another (Figure 1, panel A). Vaccine and wild-type strains were generally discriminated by lineage-specific hydrolysis probes within a single tube reaction, incapable of detecting the heterologous lineage due to high numbers of nucleotide mismatches under oligonucleotide binding sites (Figure 1, panel B) (*12*).

We selected the 2 most sensitive single- and dual-target assays on the basis of preliminary experiments using full viral RNA of wild-type and vaccine strains (Table; Technical Appendix Figure 2). For assay validation and quantification, we designed 2 in vitro transcripts (IVTs) based on the vaccine strain 17DD and an outbreak strain from Brazil (*5*), as described previously (*12*). 

**Table:** Oligonucleotides for new yellow fever virus real-time RT-PCRs*

Oligonucleotide name	Primer/probe†	Sequence, 5′ → 3′‡	Target genomic domain, no. bases	Orientation
Single-target assay				
YFVsingle-fwd	Primer	GTGGAGRAGCAGRGCRGATGAG	2,653–2,674	+
YFVsingle-rv	Primer	AAHGGRTGWGTYCCTCTCTGR	2,743–2,763	-
YFVsingleP-vac	Probe (FAM)	TTCTGTTGTCGTGCAGGATCCAAAGAATG	2,710–2,738	+
YFVsingleP-wt	Probe (YAK)	TAGAYATYTCAGTGGTGGTYCAAGACYC	2,703–2,730	+
Dual-target assay				
YFVdual-fwd-vac	Primer	GGGACTAGCGTGATCATTGA	3,296–3,315	+
YFVdual-rv-vac	Primer	GAATAACTTTCCCGCTATCCGT	3,356–3,377	-
YFVdualP-vac	Probe (FAM)	TCCCCGTCCATCACAGTTGCC	3,317–3,337	-
YFVdual-fwd-wt	Primer	CAATGCCATYCTTGAGGAGAAT	2,677–2,698	+
YFVdual-rv-wt	Primer	CGGATGTGTCCCTCTCTG	2,744–2,761	-
YFVdualP-wt	Probe (YAK)	TCTTGRACCACCACTGAGATGTCTACC	2,701–2,727	-

*25 µL real-time RT-PCR reactions were performed using the Superscript III one-step RT-PCR system with Platinum Taq polymerase (Thermo Fisher Scientific, Darmstadt, Germany). Target genomic domain positions according to GenBank reference genome NC_002031. Reactions were set up with 5 μL of RNA; 12.5 μL of 2× reaction buffer; 0.4 μL of a 50 mM magnesium sulfate solution (Superscript III one-step RT–PCR system with Platinum Taq polymerase kit, Thermo Fisher Scientific); 1 μg of nonacetylated bovine serum albumin; and 1 µL enzyme. Single-target assay reactions contained 400 nM forward primer, 600 nM reverse primer, and 280 nM of each probe. Dual-target assay reactions contained 400 nM of each primer and 220 nM of each probe. RT-PCR, reverse transcription PCR. †Probes are labeled with either fluorescein amidite (FAM) or Yakima Yellow (YAK) at the 5′-end and a Black Hole Quencher (TIB MOLBIOL Syntheselabor GmbH, Berlin, Germany) at the 3′-end. Primer concentrations were optimized using the YFV vaccine strain IVT and the wild-type YFV IVT. Amplification involved 50°C for 15 min, followed by 95°C for 3 min and 45 cycles of 95°C for 15 s and 58°C for 30 s with fluorescence read at the 58° annealing/extension step on a LightCycler 480 thermocycler (Roche, Basel, Switzerland). ‡H = A/C/T, M = A/C, R = A/G, W = A/T, Y = C/T.

The 95% lower limit of detection of the single- and dual-target assays ranged from 4.0 to 8.8 RNA copies/reaction for vaccine and wild-type YFV strains (Technical Appendix Figure 3). Discrimination between vaccine and wild-type strains was reliable even at high concentrations of IVTs and full viral RNA in the range of 10^6^ copies/reaction. Assay specificity was assessed using a set of 39 high-titer flavivirus cell culture isolates (Technical Appendix Figure 1, panel A), all of which tested negative in the novel assays.

**Figure 2:**
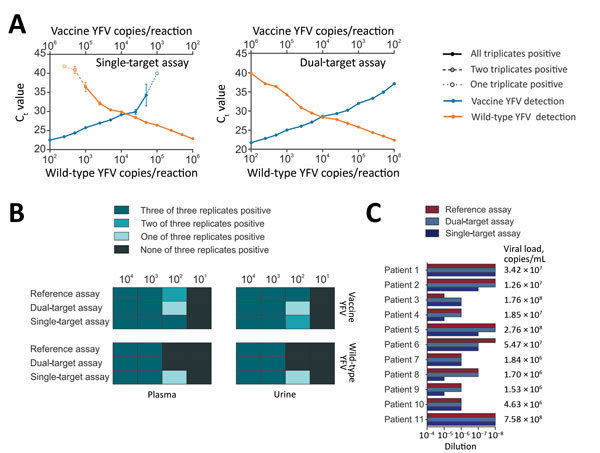
Validation of new real-time RT-PCRs for differentiation between vaccine and wild-type YFV. A) Effects of target competition on YFV real-time RT-PCRs. Mean cycle threshold (C_t_) values are plotted against IVT concentrations. Triplicates were tested for each datum point. B) Validation of the assays with clinical matrices. Spiked viruses were vaccine strain 17D and the American genotype 2 wild-type strain BOL88/1999. RNA purification was performed using the MagNA Pure 96 Viral NA Small Volume Kit (Roche, Basel, Switzerland) according to the manufacturer’s instructions. C) Clinical validation. Clinical specimens (serum, liver, whole blood, and plasma) from 11 YFV-infected patients were tested. RNA was extracted using the MagMAX Pathogen RNA/DNA Kit (Thermo Fisher, São Paulo, Brazil) and serial dilutions of the RNA were tested using the new assays and a YFV reference assay (*12*). Viral loads were determined for clinical specimens using a commercially available quantitative real-time RT-PCR (Bio Gene Research Yellow Fever PCR kit; Bioclin, Minas Gerais, Brazil), following the manufacturer´s instructions. Standard curves and sample copies per millileter were calculated using an in-house IVT standard. IVT, in vitro transcript; RT-PCR, reverse transcription PCR; YFV, yellow fever virus.

Hypothetically, near-simultaneous infection with wild-type YFV and vaccination may occur in the outbreak setting in Brazil. In the case of co-occurrence of vaccine and wild-type YFV within a single sample, 1 target may occur in relatively higher concentrations than, and thus outcompete amplification of, the other target, resulting in an incomplete test result. We observed no target competition with the dual-target assay even in the presence of high concentrations of the heterologous RNA (Figure 2, panel A). In contrast, the single-target assay showed decreased sensitivity at <1,000 copies/reaction upon the presence of 100–500-fold higher concentrations of the heterologous target.

Commonly used clinical specimens for YFV diagnostics may contain substances that can interfere with PCR (*13*). To assess our assays’ performance in different clinical matrices, we used human plasma and urine previously tested negative for YFV and spiked them with 10^1^–10^6^ copies/mL of either vaccine or wild-type YFV. Three replicates of each spiked specimen were purified individually and tested by using our PCRs and a YFV reference assay (*13*). We detected samples containing >1,000 copies/mL in all replicates irrespective of the clinical matrix (Figure 2, panel B). Detection of samples containing <100 copies/mL was unreliable in all 3 assays. As exemplified before for Zika virus, final RNA copy numbers in eluates used for RT-PCR will depend on the RNA extraction protocol, illustrating that even assays with analytical sensitivity in the single-copy range may not correctly detect weakly positive clinical specimens (*12*).

Clinical specimens may differ from spiked materials, and assay performance needs to be assessed in an outbreak context. Therefore, we compared the new assays to the reference assay (*13*) in a Brazilian flavivirus reference laboratory, using different clinical specimens obtained from 11 YF cases previously confirmed by nucleotide sequencing as wild-type YFV infections. The sensitivity of the dual-target assay was identical to that of the reference assay, whereas the single-target assay was slightly less sensitive (Figure 2, panel C). Identification of wild-type YFV was reliable in all cases, consistent with specific detection of lineages even in highly positive clinical specimens.

## Conclusions

The new PCRs we describe enable YFV detection with diagnostic sensitivity. The dual-target assay was superior to the single-target assay in sensitivity and robustness to target competition. However, the single-target assay may be advantageous in resource-limited settings and may be more convenient for multiplex usage in combination with assays targeting co-circulating arboviruses, such as chikungunya, Zika, and dengue viruses. Beyond rapid test results, the real-time RT-PCR–based protocols provide considerably higher sensitivity than protocols aiming at generating longer PCR amplicons necessary for strain discrimination by nucleotide sequencing, enabling conclusive results even when virus concentrations in specimens are low or when these materials are available only in limited quantity. Of note, PCR-based YFV detection is most reliable 5–7 days after symptom onset, during the viremic phase. Reliable YFV surveillance should thus include serologic methods. However, serologic tests used for virologic diagnostics cannot discriminate between vaccination and wild-type YFV infection.

Of note, our novel assays are limited to vaccine and American YFV wild-type strains. West African wild-type stains would be detected by our YFV vaccine assays due to the close genetic relatedness between these strains, but our assays are not suitable to detect the genetically diverse Eastern and Central African wild-type strains. If needed, one could extend our assays by an additional primer/probe combination targeting the Eastern and Central African genotypes.

Recently, the first attenuated live dengue virus vaccine was approved in several countries, including Brazil (*14*). Inactivated Japanese encephalitis virus vaccines are currently replaced by attenuated live vaccines in Asia (*15*), and an attenuated live West Nile virus vaccine has completed a phase II clinical trial (*16*). Large-scale deployment of these vaccines will raise the need to discriminate between potential vaccination-associated events and wild-type virus infection in symptomatic patients. Our work with YFV may provide a diagnostic blueprint for establishing and validating suitable methods for differentiating between vaccine and wild-type viruses for these other viruses as well.

Technical Appendix Additional information about yellow fever virus surveillance, Brazil, 2017.
