# Supplementary material for: Lineage-Specific Real-Time RT-PCR for Yellow Fever Virus Outbreak Surveillance, Brazil
Source: Emerg Infect Dis. 2017 Nov;23(11):1867–71. doi: 10.3201/eid2311.171131 (PMC5652426; doi:10.3201/eid2311.171131)
Supplement: Technical Appendix — Additional information about yellow fever virus surveillance, Brazil, 2017. [file 17-1131-Techapp-s1.pdf]

Article DOI: https://doi.org/10.3201/eid2311.171131

Lineage-Specific Real-Time RT-PCR for Yellow Fever Virus Outbreak Surveillance, Brazil

Technical Appendix

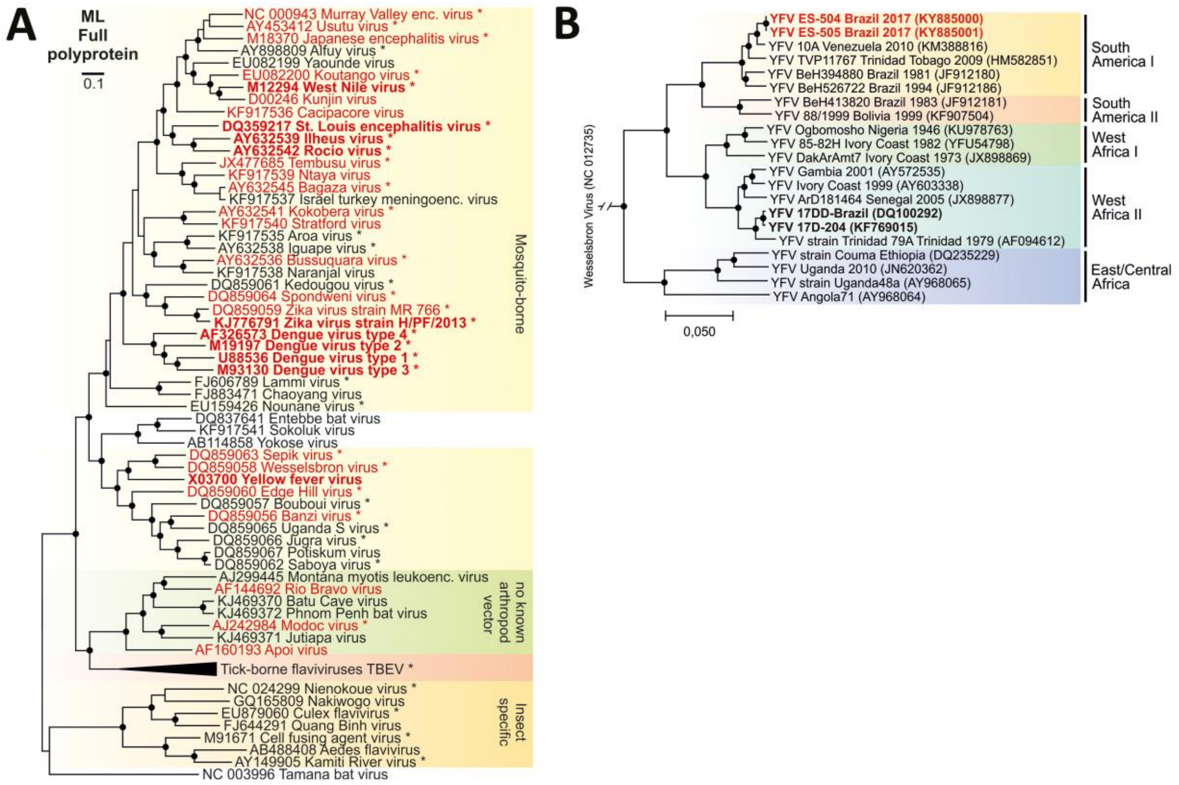

Technical Appendix Figure 1. Genetic relationships of flaviviruses and yellow fever virus genotypes. A) Maximum-likelihood phylogeny of the full polyprotein genes of selected flaviviruses. Red indicates human pathogenic viruses. Bold indicates viruses of medical importance in Brazil. Asterisks indicate viruses that were used for specificity testing of real-time RT-PCR assays. The scale bar indicates evolutionary distances of 0.1 differences per site. The HKY nucleotide substitution model was used with a complete deletion option in MEGA7. Black circles at nodes represent over 75% statistical support of grouping from 1,000 bootstrap replicates. B) Neighbor-joining phylogeny of complete coding sequences of representative Yellow Fever virus strains identified by strain name, country, year of isolation (where applicable) and GenBank accession number. Bold and red type indicates sequences of the current outbreak in Brazil (*5*). Bold indicates vaccine strains. The tree was generated using a percentage distance substitution model in MEGA7 and the complete deletion option. The final dataset encompassed 11,139 nt. Black circles at nodes represent over 98% support of grouping from 1,000 bootstrap replicates. The scale bar indicates genetic distance of 5%. The tree was rooted by Wesselsbron virus (branches truncated as indicated by slashed lines).

Supplementary Figure S1. Bench protocol for real-time RT-PCR assays

CHARITÉ CAMPUS MITTE

CharitéCentrum für diagnostische und präventive Labormedizin

Charité | Campus Mitte | 10098 Berlin

Institut für Virologie
-HELMUT-RUSKA-HAUS-
Univ.-Prof. Dr. med. J. F. Drexler

Tel. +49-30-450-52 50 92
Fax +49-30-450-752 59 07
felix.drexler@charite.de
http://virologie-ccm.charite.de/

Development and clinical validation of lineage-specific real-time RT-PCR assays for Yellow Fever virus surveillance

Single-target and dual-target assay

Example formulation: Thermo Fisher SuperScriptIII OneStep RT-PCR System with Platinum Taq DNA Polymerase

Single target assay

MasterMix	single rxn [µL]
H_2O (RNAse free)	1.2
$MgSO_4$(50mM)	0.4
2x Reaction mix	12.5
BSA (1 mg/ml)*	1
Fwd primer (10 µM)	1
Rev primer (10 µM)	1.5
Probe 1 (10 µM)	0.7
Probe 2 (10 µM)	0.7
SSIII/Taq EnzymeMix	1
	20
Template RNA	5

Dual target assay

MasterMix	single rxn [µL]
H_2O (RNAse free)	-
$MgSO_4$(50mM)	0.4
2x Reaction mix	12.5
BSA (1 mg/ml)*	1
Fwd primer 1 (10 µM)	1
Fwd primer 2 (10 µM)	1
Rev primer 1 (10 µM)	1
Rev primer 2 (10 µM)	1
Probe 1 (10 µM)	0.55
Probe 2 (10 µM)	0.55
SSIII/Taq EnzymeMix	1
	20
Template RNA	5

*non-acetylated. This component is only necessary if using glass capillary LightCycler. Can be replaced with water in plastic vessel machines such as ABI 7500, LC 480, etc.

Thermocycling conditions

50°C	15′	
95°C	3′	
95°C	15″	45x
58°C	30″	45x
40°C	30″	

Primers/probe (5‘-3‘)

YFVsingle-fwd	GTGGAGRAGCAGRGCRGATGAG
YFVsingleP-vac	6-FAM-TTCTGTTGTCGTGCAGGATCCAAAGAATG-BBQ
YFVsingleP-wt	YAK-TAGAYATYTCAGTGGTGGTYCAAGACYC-BBQ
YFVsingle-rv	AAHGGRTGWGTYCCTCTCTGR
YFVdual-fwd-vac	GGGACTAGCGTGATCATTGA
YFVdualP-vac	6FAM-TCCCCGTCCATCACAGTTGCC-BBQ
YFVdual-rv-vac	GAATAACTTTCCCGCTATCCGT
YFVdual-fwd-wt	CAATGCCATYCTTGAGGAGAAT
YFVdualP-wt	YAK-TCTTGRACCACCACTGAGATGTCTACC-BBQ
YFVdual-rv-wt	CGGATGTGTCCCTCTCTG

Technical Appendix Figure 2. Bench protocol for real-time RT-PCR assays.

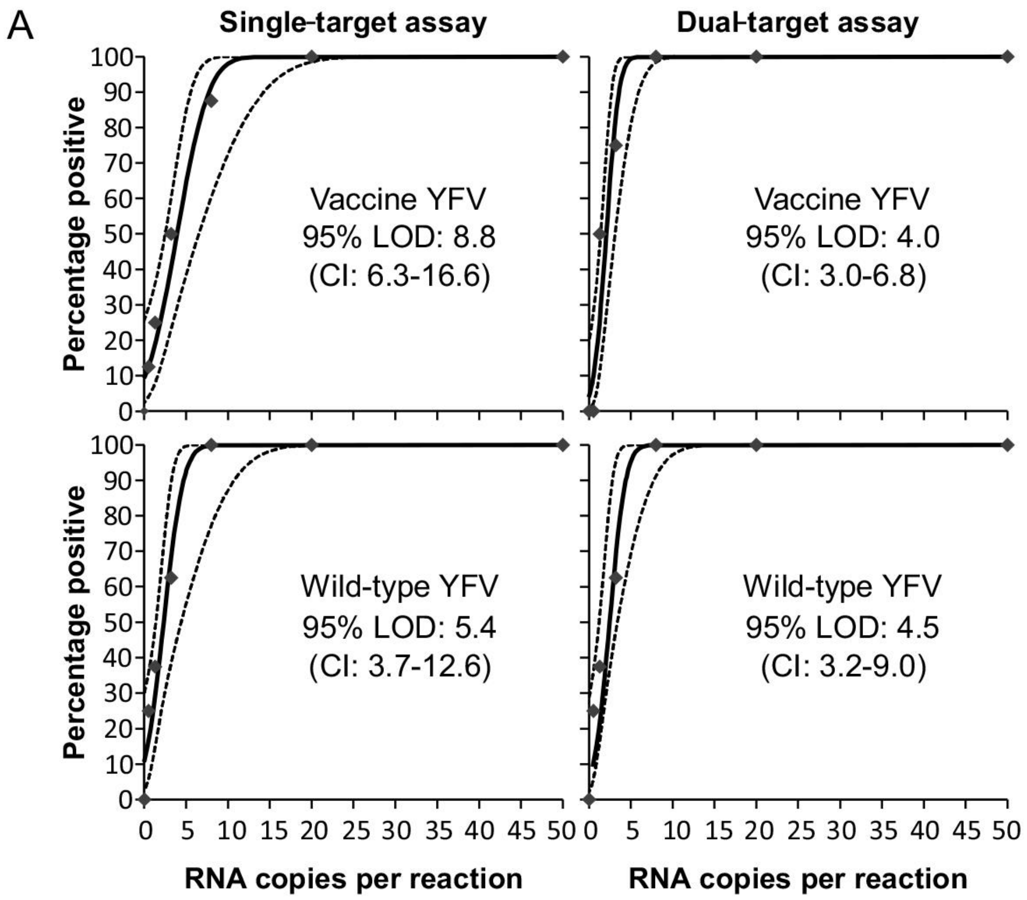

Technical Appendix Figure 3. Analytical sensitivity of YFV real-time RT-PCR assays. CI: confidence interval; LOD: lower limit of detection. Probability of detection determined in probit regression analyses is plotted against IVT copies/reaction in 8 replicates. Diamonds represent observed positive results. Solid line shows predicted proportion of positive results at a given IVT input; dashed line shows 95% CI. In-vitro transcripts were designed upon strain 17DD and the Brazilian outbreak strain ES-505, GenBank accession numbers DQ100292 and KY885001, respectively.
